# Supplementary material for: REFLACX, a dataset of reports and eye-tracking data for localization of abnormalities in chest x-rays
Source: Sci Data. 2022 Jun 18;9:350. doi: 10.1038/s41597-022-01441-z (PMC9206650; doi:10.1038/s41597-022-01441-z)
Supplement: Supplementary file 2 — Supplementary File 1 [file 41597_2022_1441_MOESM2_ESM.pdf]

Instruction Document

Version 02

NIH NIBIB R21: A scalable non-intrusive image annotation method using eye tracking for training deep learning models in radiology (PI: Tasdizen; Co-I: Schroeder, Srikumar, Drew, Auffermann)

PhD project: Bigolin Lanfredi

Radiology readers: Schroeder, Auffermann, Duong, Chan, Mann

Images: MIT MIMC-CXR database[1]

Support devices

Abnormal mediastinal contour
Enlarged cardiac silhouette
Enlarged hilum
Hiatal hernia

Pneumothorax
Pleural abnormality

Consolidation
Groundglass opacity
Atelectasis
Lung nodule or mass
Pulmonary edema
High lung volume / emphysema
Interstitial lung disease

Acute fracture

Other

Chest radiograph labels:

Labels are grouped in a single list in the research workstation menu in the order shown, approximating the grouping used in radiology report dictation templates.

Support devices

Abnormal mediastinal contour
Enlarged cardiac silhouette
Enlarged hilum
Hiatal hernia

Pneumothorax
Pleural abnormality

Consolidation
Groundglass opacity
Atelectasis
Lung nodule or mass
Pulmonary edema
High lung volume / emphysema
Interstitial lung disease

Acute fracture

Other

Select the Support devices label to indicate that support devices are present. We will not draw bounding boxes on support devices. The Support devices label merely indicates their presence. Examples of support devices are:

- Endotracheal tubes
- Central venous catheters
- Pulmonary artery catheters
- Pacemakers
- Pleural or mediastinal drains
- Gastric or feeding tubes

Support devices

Abnormal mediastinal contour
Enlarged cardiac silhouette
Enlarged hilum
Hiatal hernia

Pneumothorax
Pleural abnormality

Consolidation
Groundglass opacity
Atelectasis
Lung nodule or mass
Pulmonary edema
High lung volume / emphysema
Interstitial lung disease

Acute fracture

Other

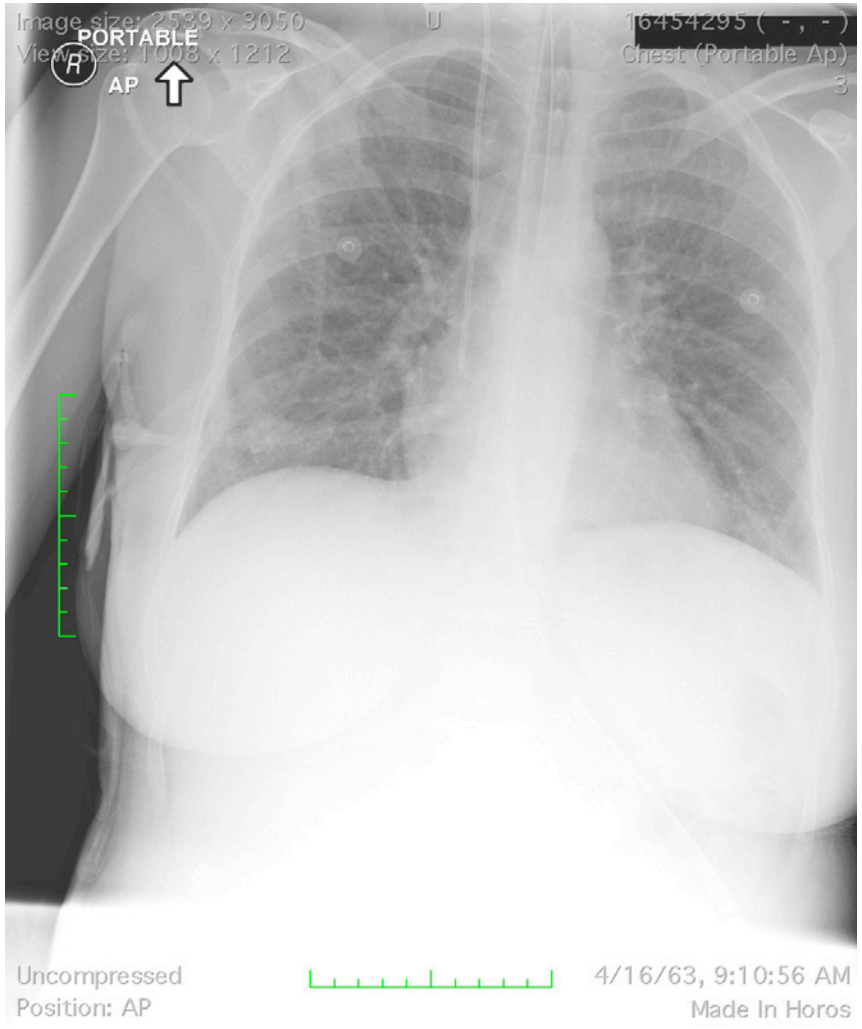

Support devices

Abnormal mediastinal contour
Enlarged cardiac silhouette
Enlarged hilum
Hiatal hernia

Pneumothorax
Pleural abnormality

Consolidation
Groundglass opacity
Atelectasis
Lung nodule or mass
Pulmonary edema
High lung volume / emphysema
Interstitial lung disease

Acute fracture

Other

Representative examples of Abnormal mediastinal contour include abnormal superior mediastinal widening (e.g., vascular abnormality) and asymmetric lower mediastinal widening (e.g., pericardial cyst or mass).

Note that ‘Enlarged cardiac silhouette’ and ‘Enlarged hilum’ labels are also available.

Support devices

Abnormal mediastinal contour
Enlarged cardiac silhouette
Enlarged hilum
Hiatal hernia

Pneumothorax
Pleural abnormality

Consolidation
Groundglass opacity
Atelectasis
Lung nodule or mass
Pulmonary edema
High lung volume / emphysema
Interstitial lung disease

Acute fracture

Other

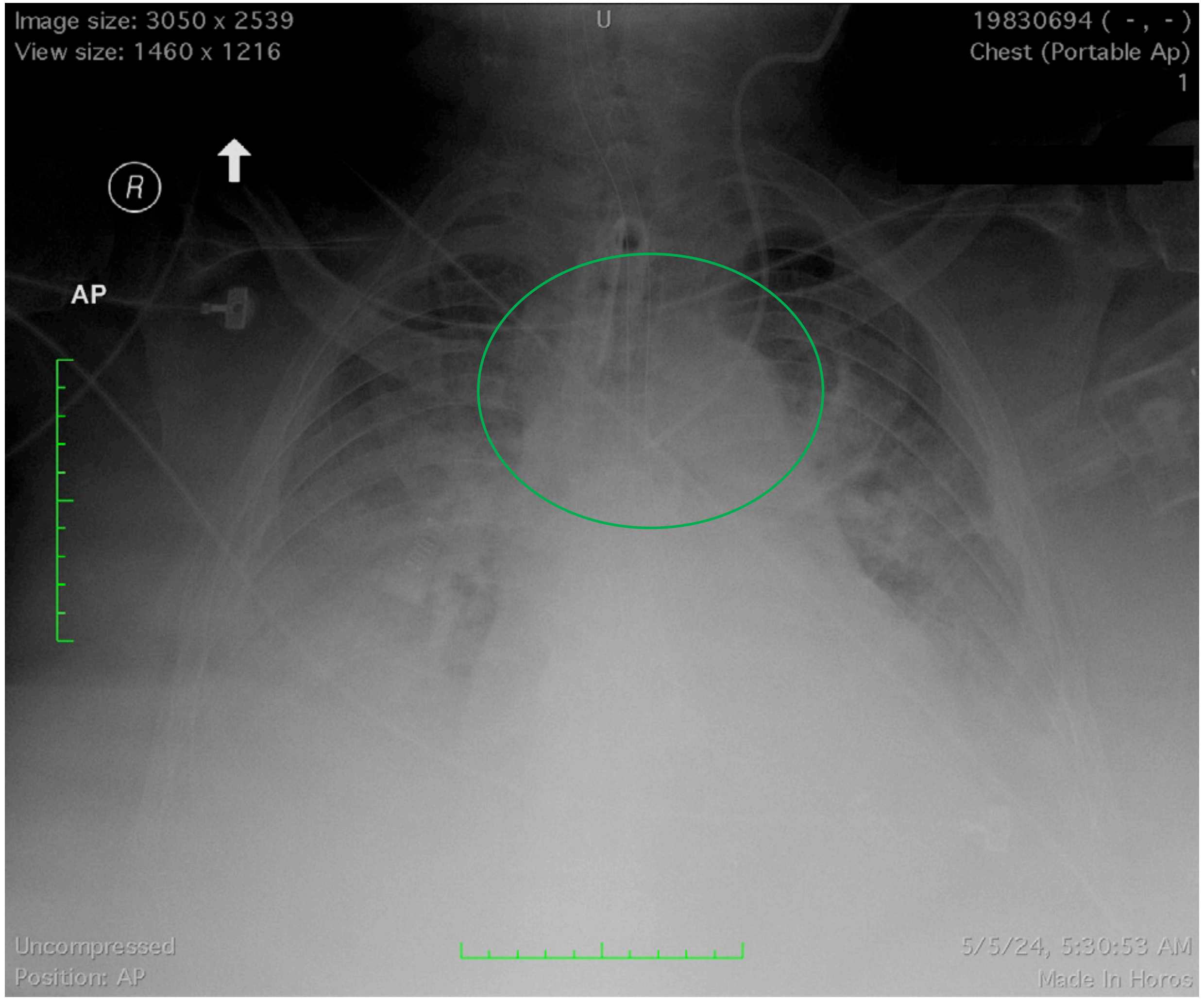

Support devices

Abnormal mediastinal contour
Enlarged cardiac silhouette
Enlarged hilum
Hiatal hernia

Pneumothorax
Pleural abnormality

Consolidation
Groundglass opacity
Atelectasis
Lung nodule or mass
Pulmonary edema
High lung volume / emphysema
Interstitial lung disease

Acute fracture

Other

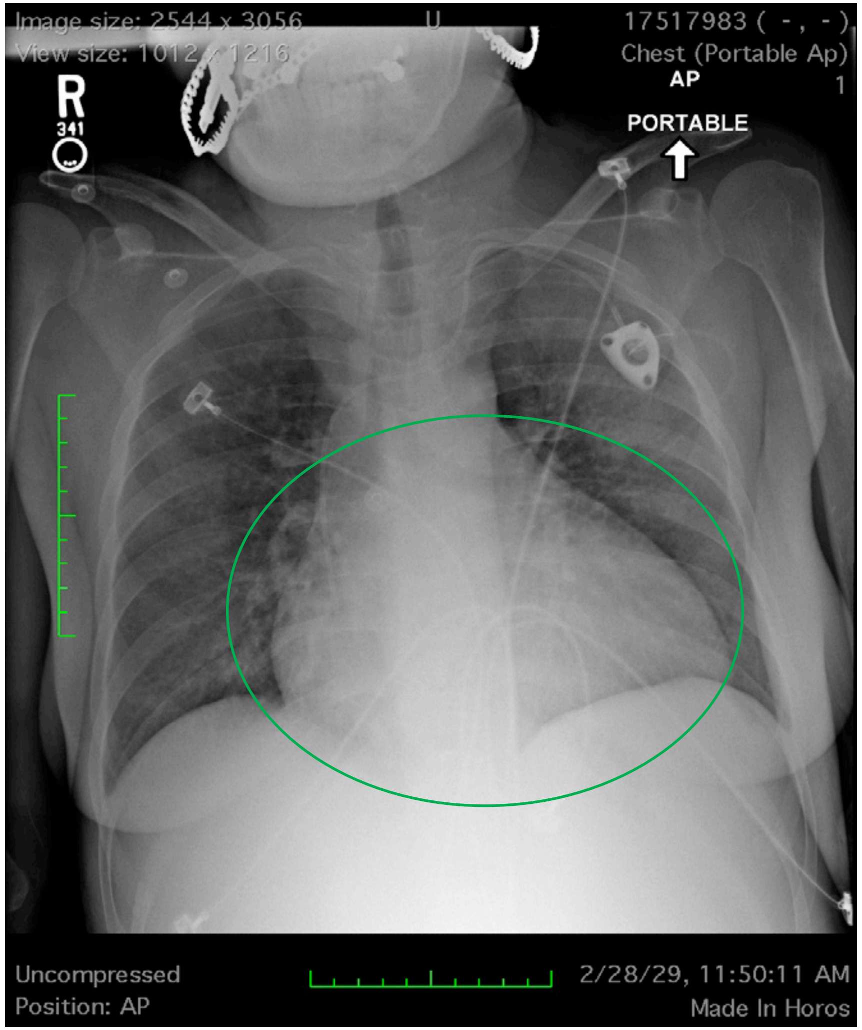

Support devices

Abnormal mediastinal contour
Enlarged cardiac silhouette
Enlarged hilum
Hiatal hernia

Pneumothorax
Pleural abnormality

Consolidation
Groundglass opacity
Atelectasis
Lung nodule or mass
Pulmonary edema
High lung volume / emphysema
Interstitial lung disease

Acute fracture

Other

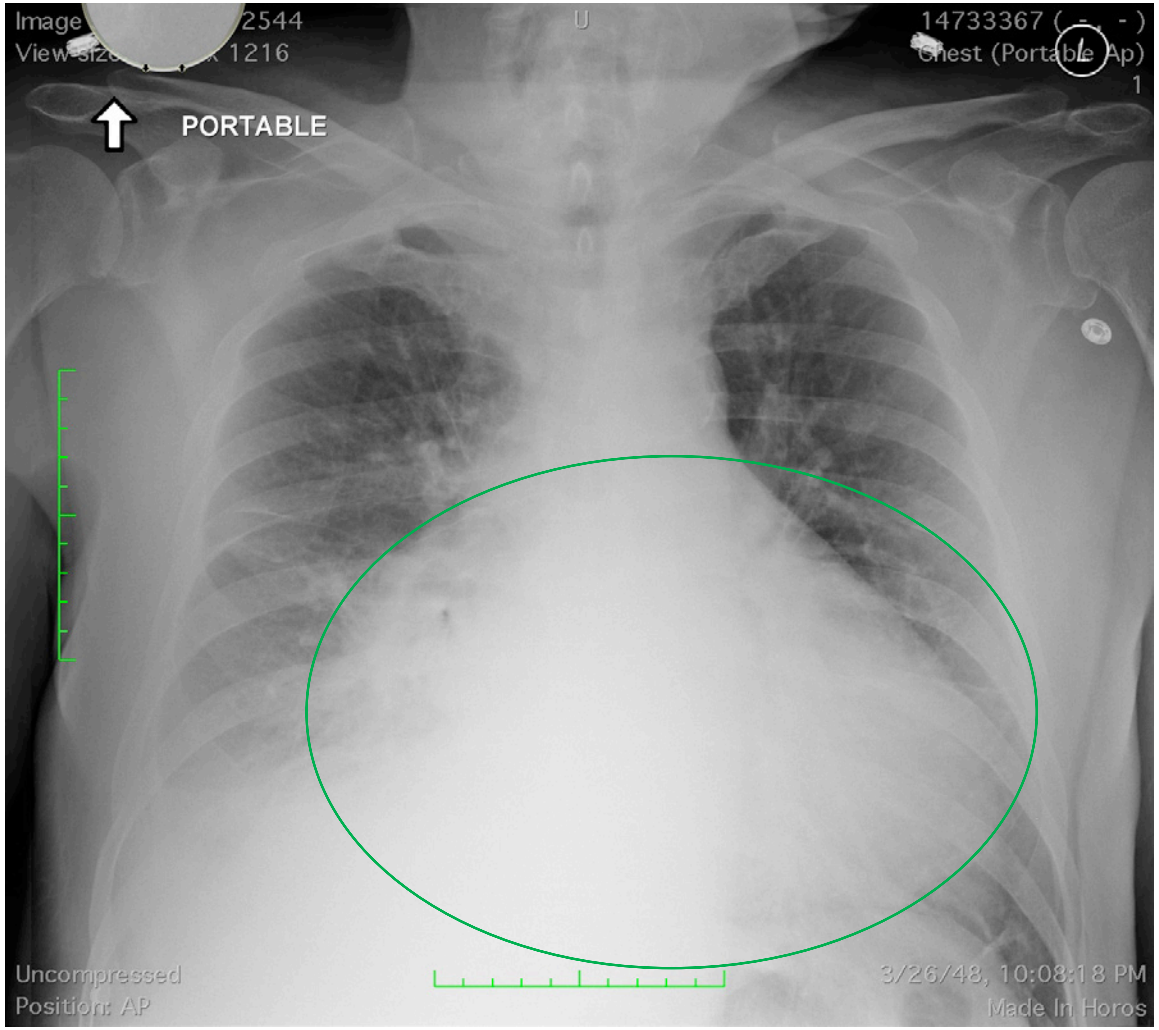

Support devices

Abnormal mediastinal contour
Enlarged cardiac silhouette
Enlarged hilum
Hiatal hernia

Pneumothorax
Pleural abnormality

Consolidation
Groundglass opacity
Atelectasis
Lung nodule or mass
Pulmonary edema
High lung volume / emphysema
Interstitial lung disease

Acute fracture

Other

Representative examples of Enlarged hilum include enlarged central pulmonary arteries, hilar lymphadenopathy (unilateral or bilateral), and hilar mass (unilateral or bilateral).

Support devices

Abnormal mediastinal contour
Enlarged cardiac silhouette
Enlarged hilum
Hiatal hernia

Pneumothorax
Pleural abnormality

Consolidation
Groundglass opacity
Atelectasis
Lung nodule or mass
Pulmonary edema
High lung volume / emphysema
Interstitial lung disease

Acute fracture

Other

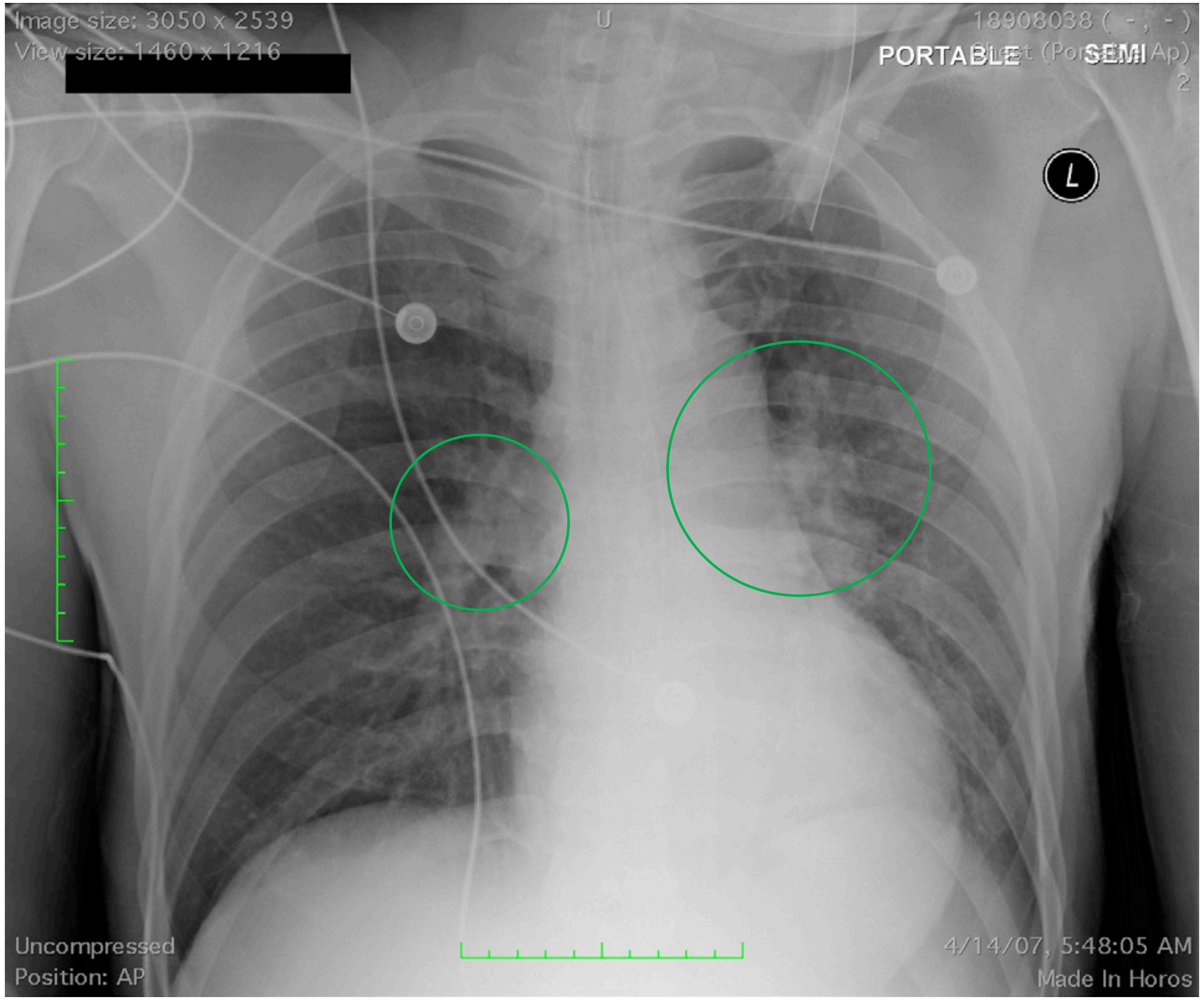

Support devices

Abnormal mediastinal contour
Enlarged cardiac silhouette
Enlarged hilum
Hiatal hernia

Pneumothorax
Pleural abnormality

Consolidation
Groundglass opacity
Atelectasis
Lung nodule or mass
Pulmonary edema
High lung volume / emphysema
Interstitial lung disease

Acute fracture

Other

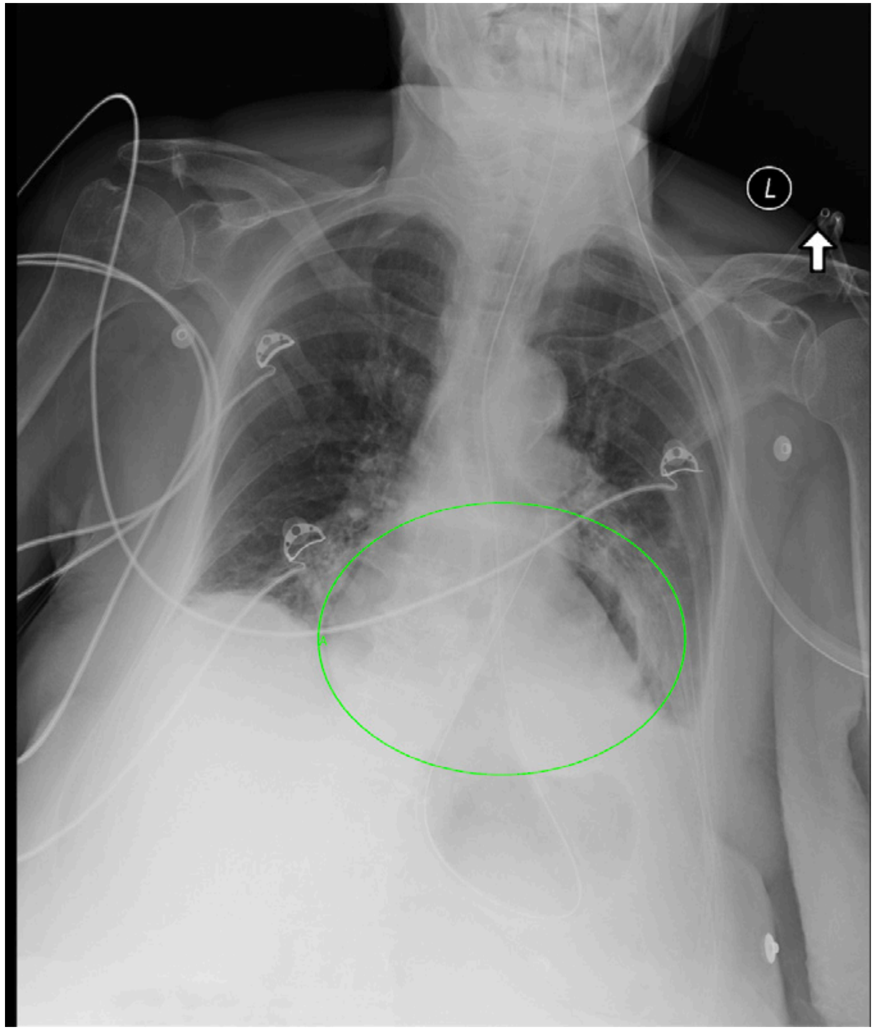

Support devices

Abnormal mediastinal contour
Enlarged cardiac silhouette
Enlarged hilum
Hiatal hernia

Pneumothorax
Pleural abnormality

Consolidation
Groundglass opacity
Atelectasis
Lung nodule or mass
Pulmonary edema
High lung volume / emphysema
Interstitial lung disease

Acute fracture

Other

For Pneumothorax, choose the bounding box to enclose the visible thin pleural line.

Support devices

Abnormal mediastinal contour
Enlarged cardiac silhouette
Enlarged hilum
Hiatal hernia

Pneumothorax
Pleural abnormality

Consolidation
Groundglass opacity
Atelectasis
Lung nodule or mass
Pulmonary edema
High lung volume / emphysema
Interstitial lung disease

Acute fracture

Other

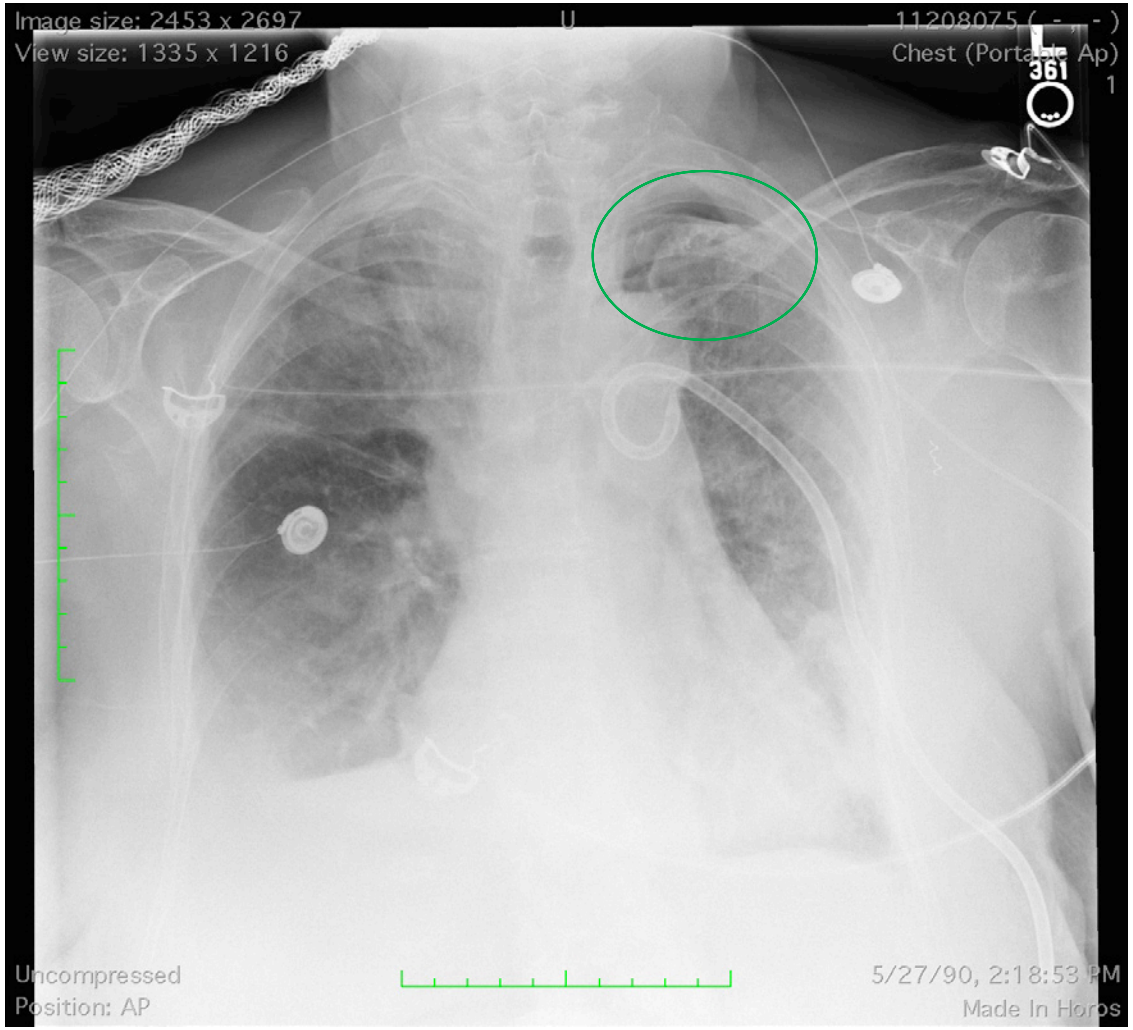

Support devices

Abnormal mediastinal contour
Enlarged cardiac silhouette
Enlarged hilum
Hiatal hernia

Pneumothorax
Pleural abnormality

Consolidation
Groundglass opacity
Atelectasis
Lung nodule or mass
Pulmonary edema
High lung volume / emphysema
Interstitial lung disease

Acute fracture

Other

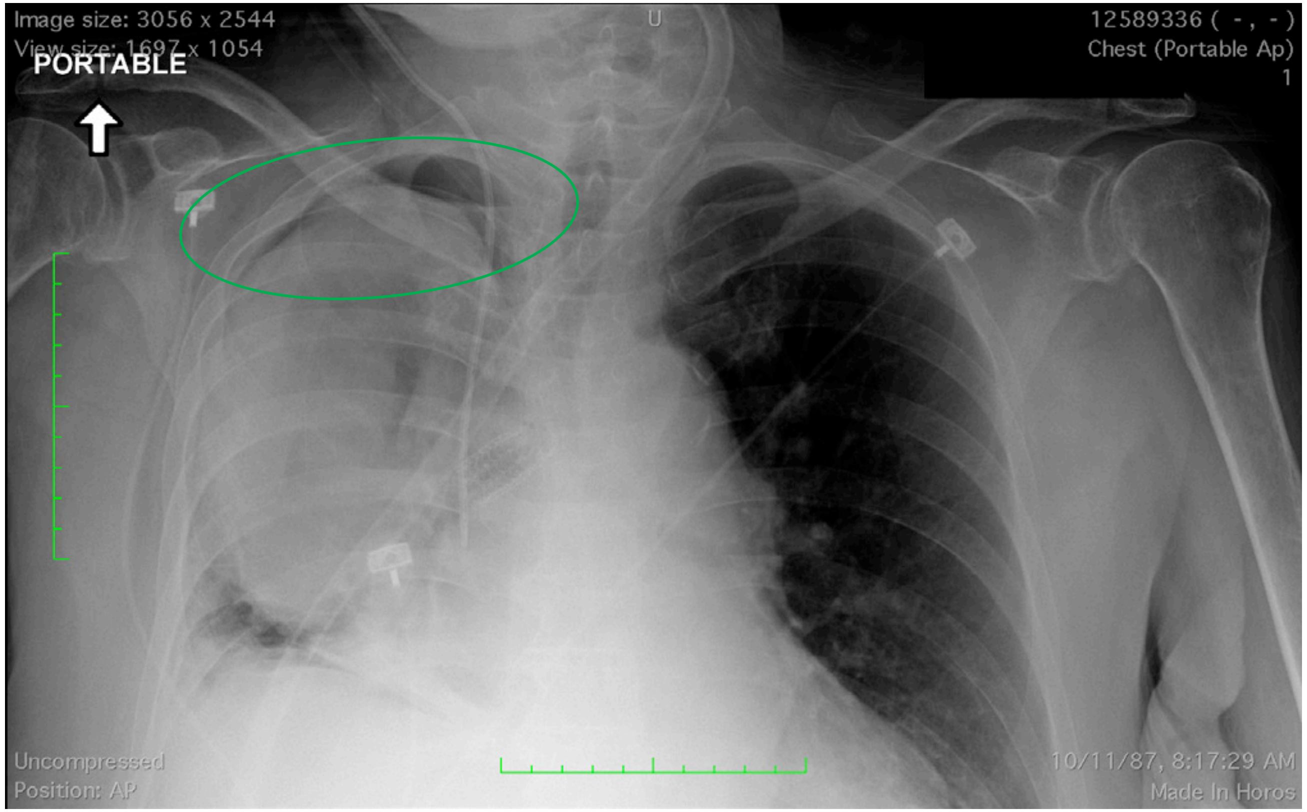

Support devices

Abnormal mediastinal contour
Enlarged cardiac silhouette
Enlarged hilum
Hiatal hernia

Pneumothorax
Pleural abnormality

Consolidation
Groundglass opacity
Atelectasis
Lung nodule or mass
Pulmonary edema
High lung volume / emphysema
Interstitial lung disease

Acute fracture

Other

Representative examples of Pleural abnormality include pleural effusion (unilateral or bilateral), pleural-based nodule or mass, and pleural calcification.

If there are CXR findings of pleural effusion, for example mild blunting of the costophrenic sulcus, then indicate Pleural abnormality, even if it is a small pleural effusion.

Support devices

Abnormal mediastinal contour
Enlarged cardiac silhouette
Enlarged hilum
Hiatal hernia

Pneumothorax
Pleural abnormality

Consolidation
Groundglass opacity
Atelectasis
Lung nodule or mass
Pulmonary edema
High lung volume / emphysema
Interstitial lung disease

Acute fracture

Other

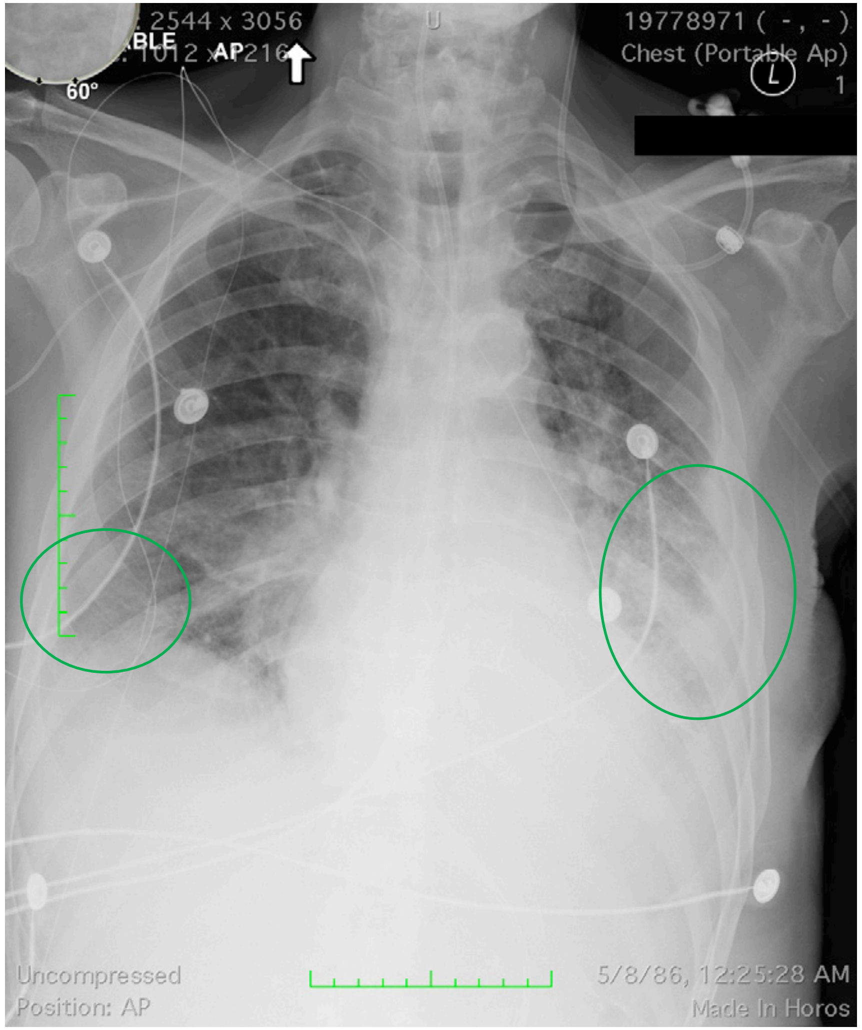

Support devices

Abnormal mediastinal contour
Enlarged cardiac silhouette
Enlarged hilum
Hiatal hernia

Pneumothorax
Pleural abnormality

Consolidation
Groundglass opacity
Atelectasis
Lung nodule or mass
Pulmonary edema
High lung volume / emphysema
Interstitial lung disease

Acute fracture

Other

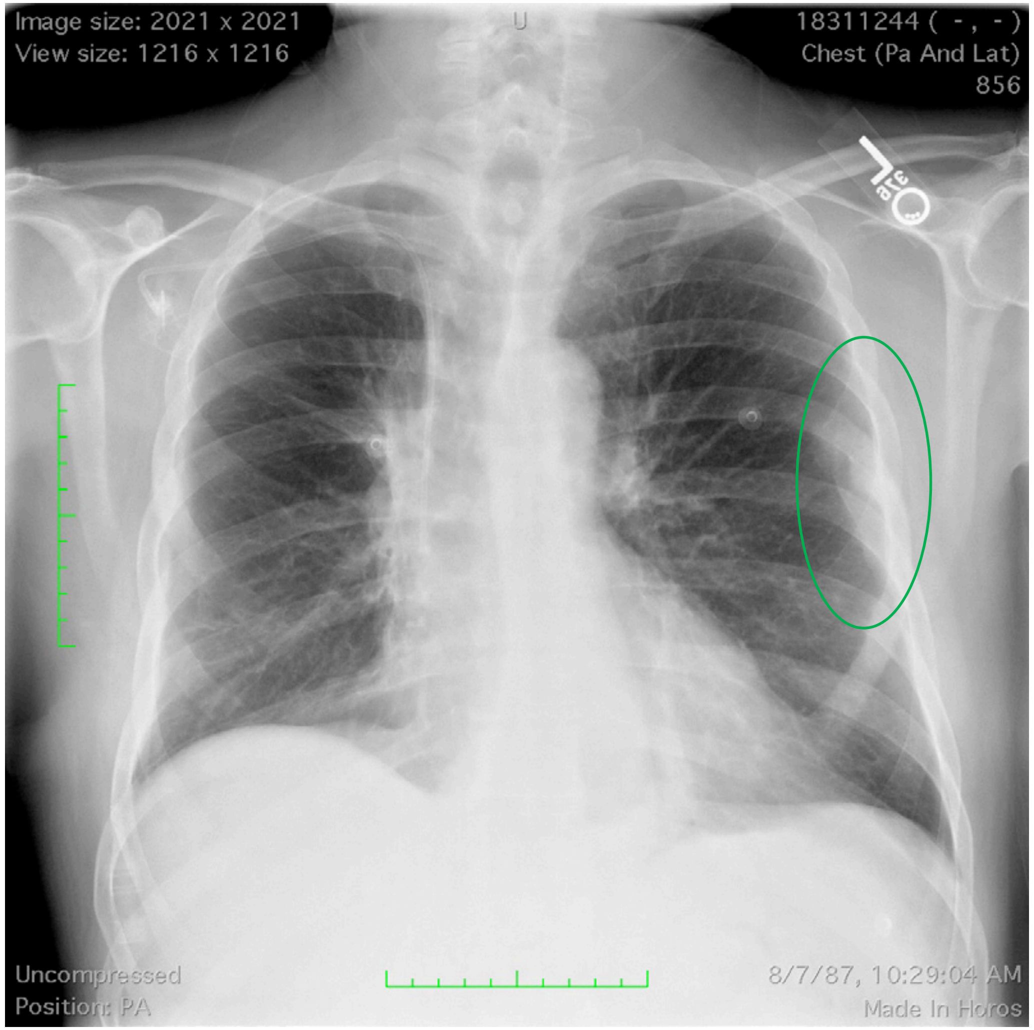

Support devices

Abnormal mediastinal contour
Enlarged cardiac silhouette
Enlarged hilum
Hiatal hernia

Pneumothorax
Pleural abnormality

Consolidation
Groundglass opacity
Atelectasis
Lung nodule or mass
Pulmonary edema
High lung volume / emphysema
Interstitial lung disease

Acute fracture

Other

Parenchymal opacification, as defined by Hansell et al. (2008)[2]

"Radiographs and CT scans—Parenchymal opacification of the lungs may or may not obscure the margins of vessels and airway walls."[2] "Consolidation indicates that definition of these margins (excepting air bronchograms) is lost within the dense opacification whereas groundglass opacity indicates a smaller increase in attenuation, in which the definition of underlying structures is preserved." [2]

"Consolidation appears as a homogeneous increase in pulmonary parenchymal attenuation that obscures the margins of vessels and airway walls. An air bronchogram may be present." [2]

Support devices

Abnormal mediastinal contour
Enlarged cardiac silhouette
Enlarged hilum
Hiatal hernia

Pneumothorax
Pleural abnormality

Consolidation
Groundglass opacity
Atelectasis
Lung nodule or mass
Pulmonary edema
High lung volume / emphysema
Interstitial lung disease

Acute fracture

Other

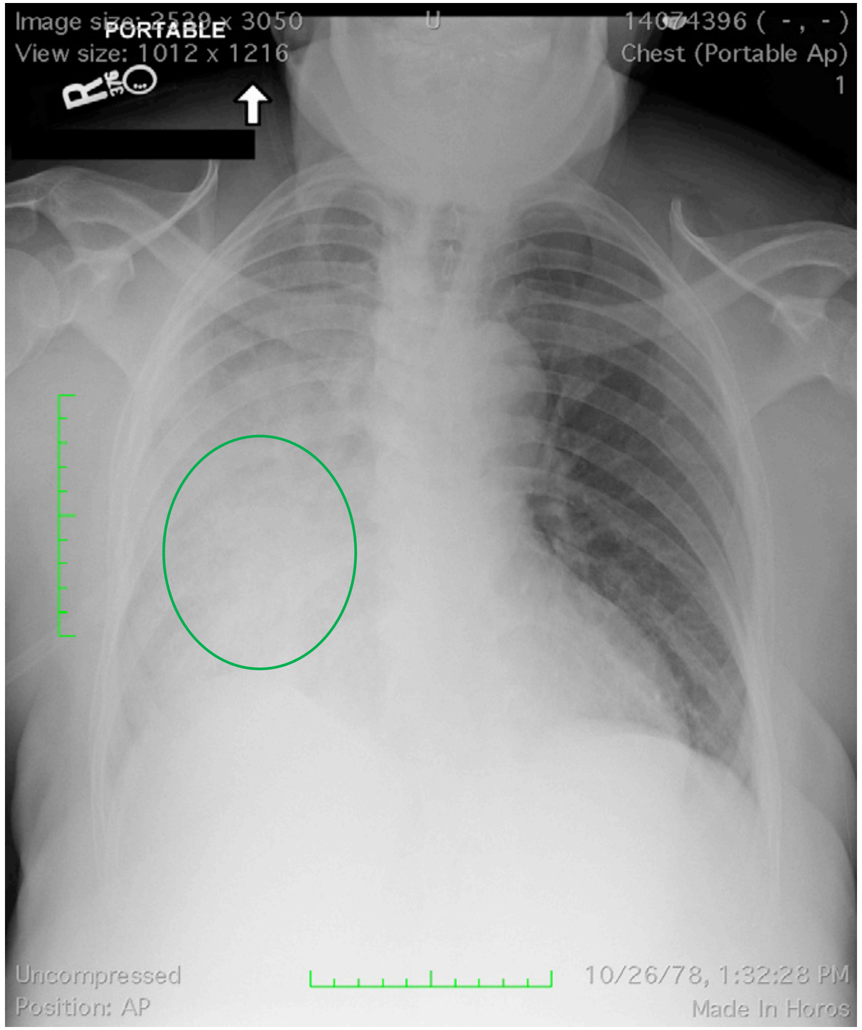

Support devices

Abnormal mediastinal contour
Enlarged cardiac silhouette
Enlarged hilum
Hiatal hernia

Pneumothorax
Pleural abnormality

Consolidation
Groundglass opacity
Atelectasis
Lung nodule or mass
Pulmonary edema
High lung volume / emphysema
Interstitial lung disease

Acute fracture

Other

Parenchymal opacification, as defined by Hansell et al. (2008)[2]

"Radiographs and CT scans—Parenchymal opacification of the lungs may or may not obscure the margins of vessels and airway walls. Consolidation indicates that definition of these margins (excepting air bronchograms) is lost within the dense opacification whereas Groundglass opacity indicates a smaller increase in attenuation, in which the definition of underlying structures is preserved." [2]

"On chest radiographs, Groundglass opacity appears as an area of hazy increased lung opacity, usually extensive, within which margins of pulmonary vessels may be indistinct." [2]

"It is caused by partial filling of airspaces." [2]

"Groundglass opacity is less opaque than consolidation, in which bronchovascular margins are obscured." [2]

Support devices

Abnormal mediastinal contour
Enlarged cardiac silhouette
Enlarged hilum
Hiatal hernia

Pneumothorax
Pleural abnormality

Consolidation
Groundglass opacity
Atelectasis
Lung nodule or mass
Pulmonary edema
High lung volume / emphysema
Interstitial lung disease

Acute fracture

Other

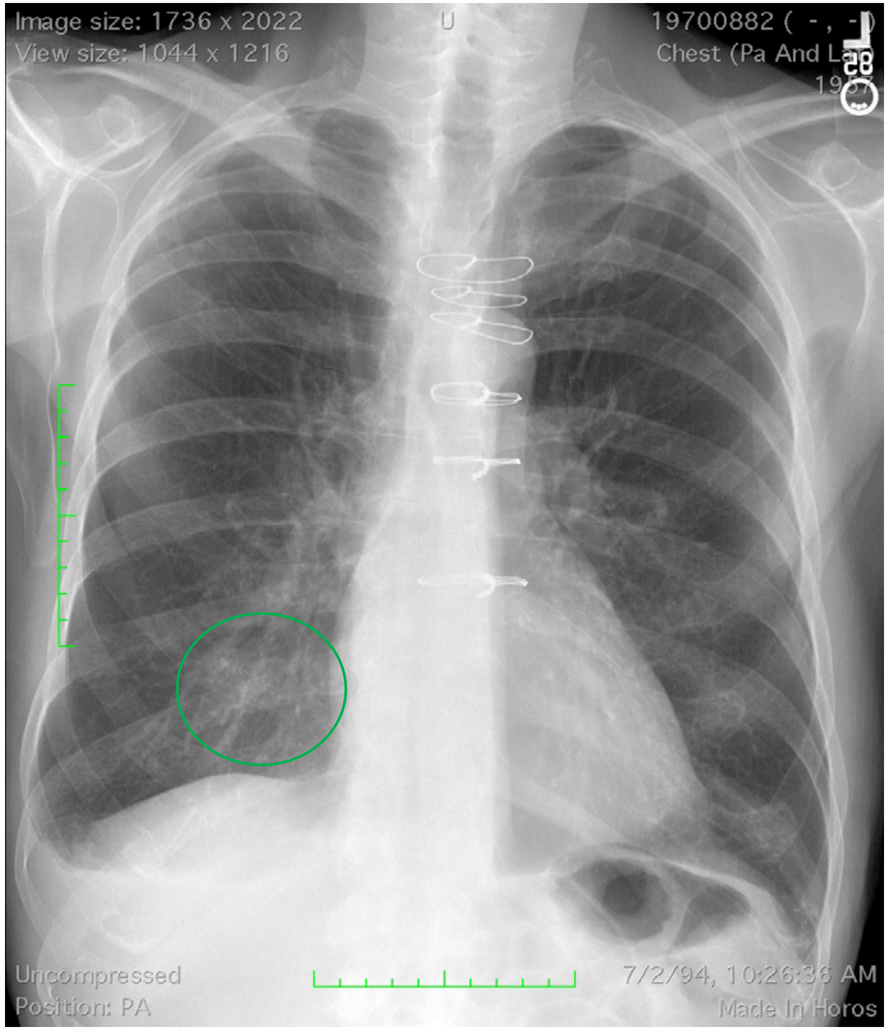

Support devices

Abnormal mediastinal contour
Enlarged cardiac silhouette
Enlarged hilum
Hiatal hernia

Pneumothorax
Pleural abnormality

Consolidation
Groundglass opacity
Atelectasis
Lung nodule or mass
Pulmonary edema
High lung volume / emphysema
Interstitial lung disease

Acute fracture

Other

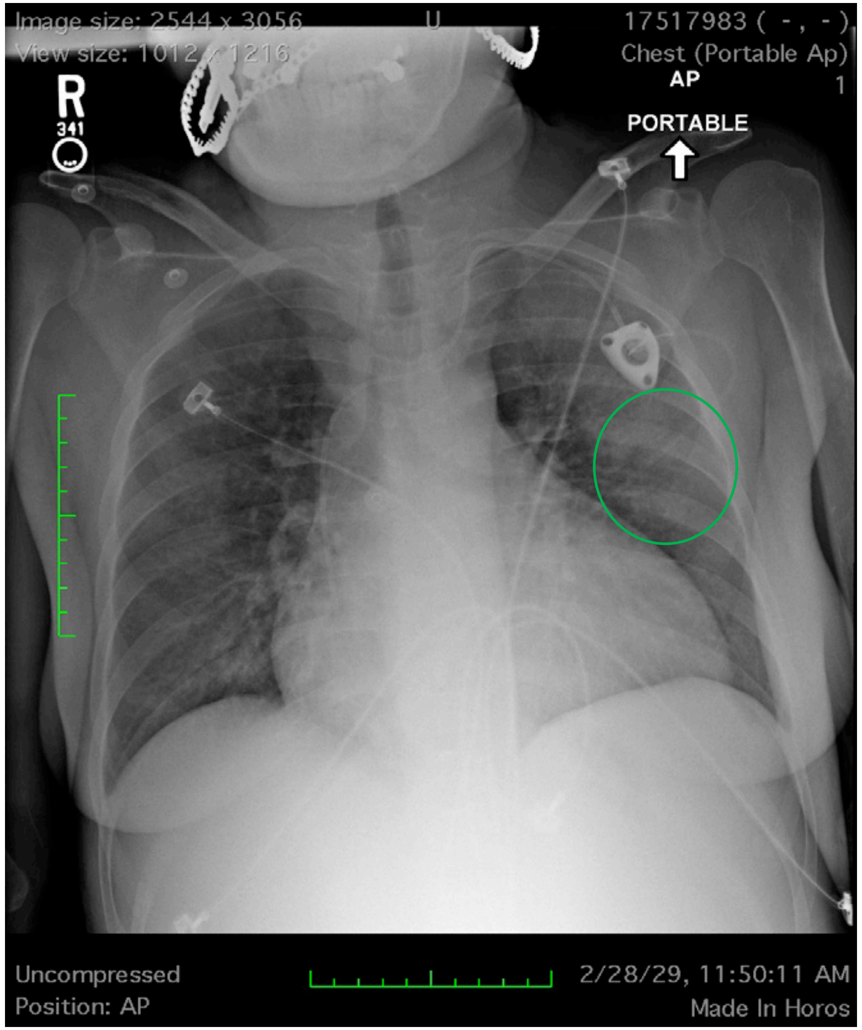

Support devices

Abnormal mediastinal contour
Enlarged cardiac silhouette
Enlarged hilum
Hiatal hernia

Pneumothorax
Pleural abnormality

Consolidation
Groundglass opacity
Atelectasis
Lung nodule or mass
Pulmonary edema
High lung volume / emphysema
Interstitial lung disease

Acute fracture

Other

For Atelectasis, do not mark mild *subsegmental* atelectasis as this is unlikely to be of clinical significance.

For Atelectasis, please do mark *segmental or multisegmental* atelectasis, as in the example in the next slide.

Support devices

Abnormal mediastinal contour
Enlarged cardiac silhouette
Enlarged hilum
Hiatal hernia

Pneumothorax
Pleural abnormality

Consolidation
Groundglass opacity
Atelectasis
Lung nodule or mass
Pulmonary edema
High lung volume / emphysema
Interstitial lung disease

Acute fracture

Other

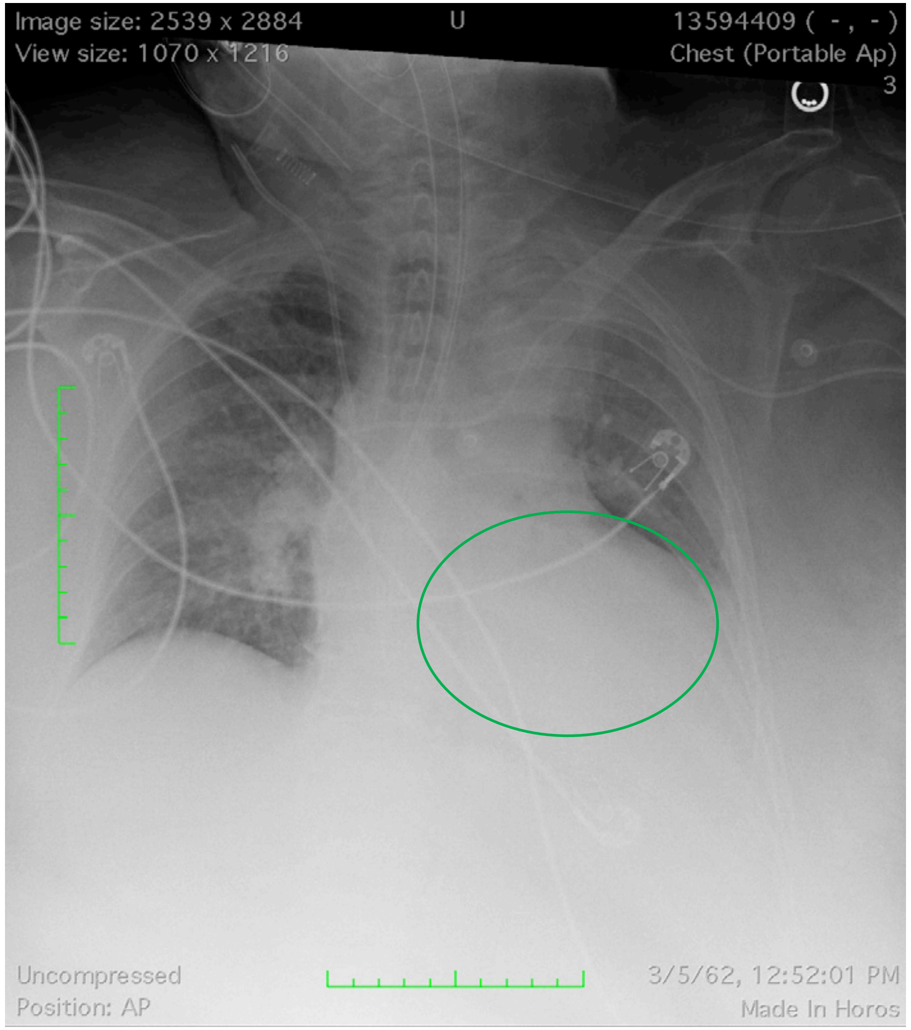

Support devices

Abnormal mediastinal contour
Enlarged cardiac silhouette
Enlarged hilum
Hiatal hernia

Pneumothorax
Pleural abnormality

Consolidation
Groundglass opacity
Atelectasis
Lung nodule or mass
Pulmonary edema
High lung volume / emphysema
Interstitial lung disease

Acute fracture

Other

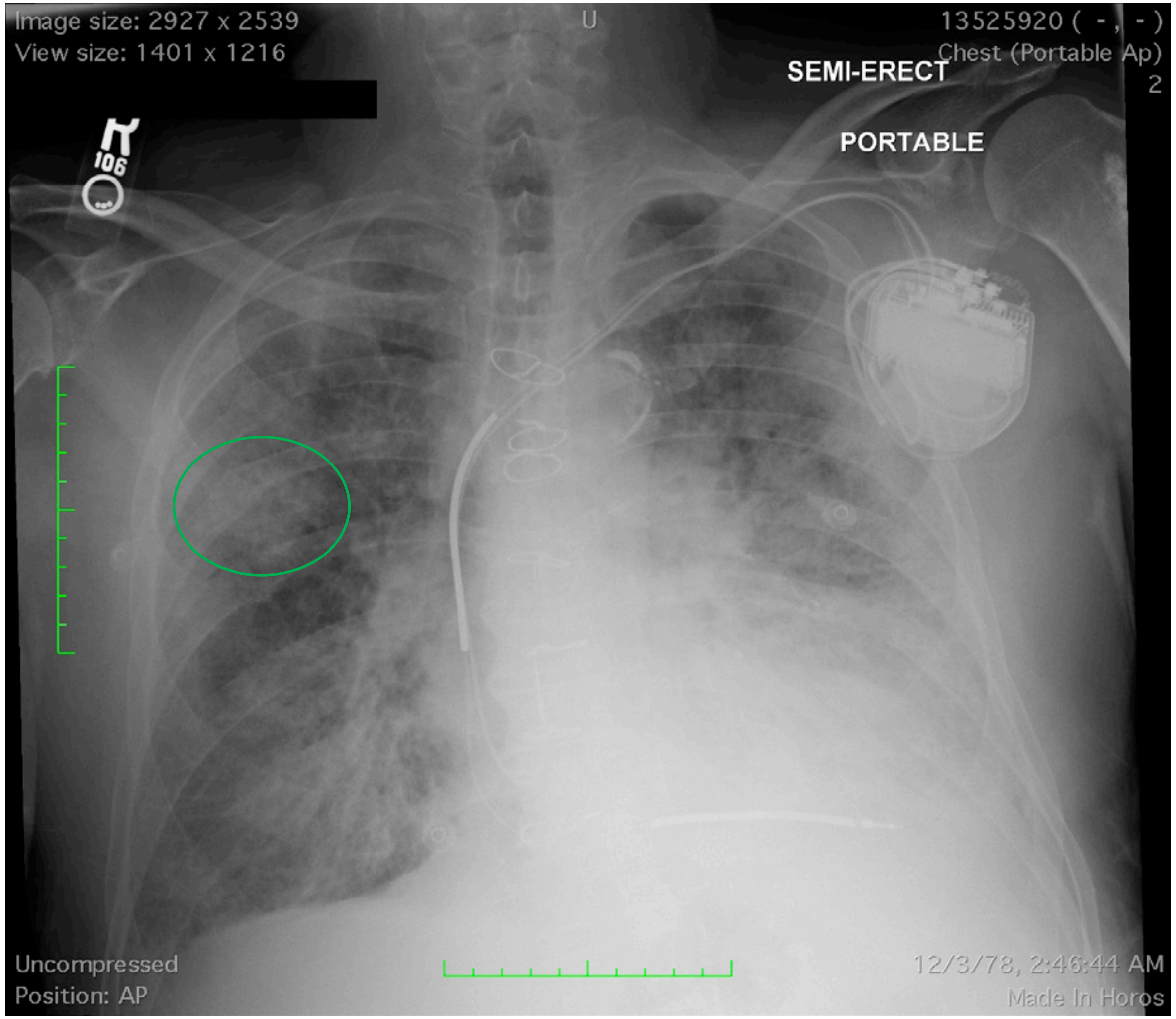

Support devices

Abnormal mediastinal contour
Enlarged cardiac silhouette
Enlarged hilum
Hiatal hernia

Pneumothorax
Pleural abnormality

Consolidation
Groundglass opacity
Atelectasis
Lung nodule or mass
Pulmonary edema
High lung volume / emphysema
Interstitial lung disease

Acute fracture

Other

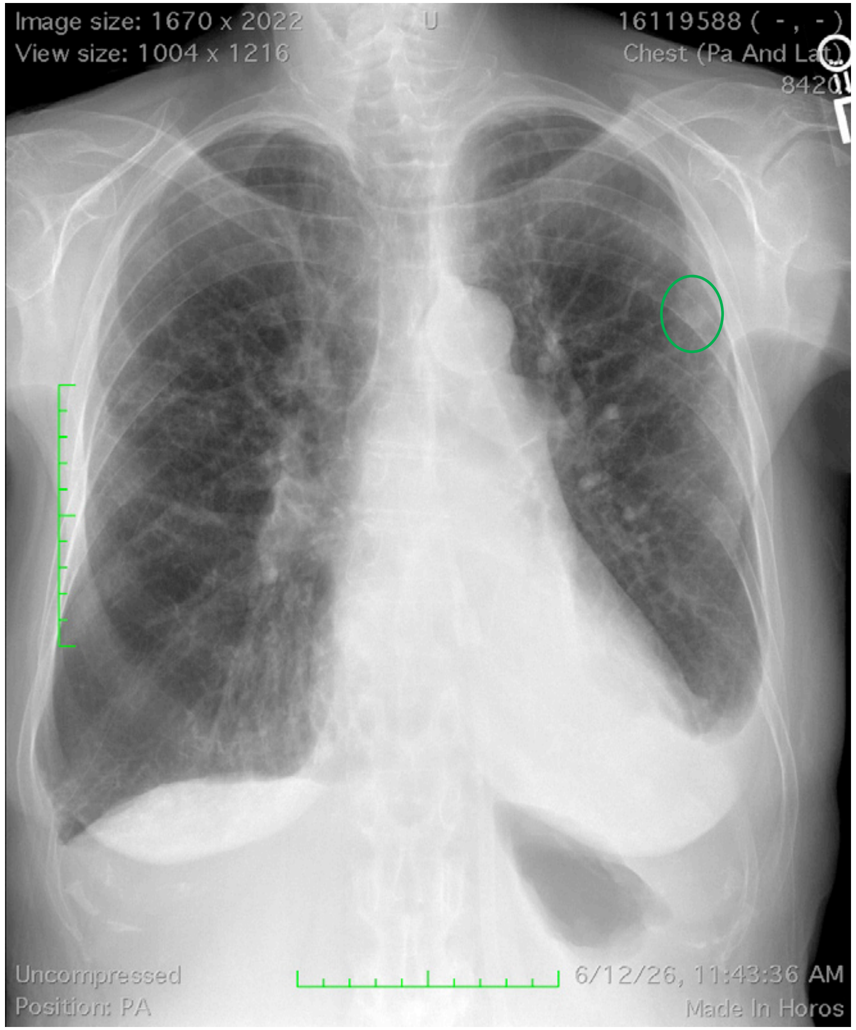

Support devices

Abnormal mediastinal contour
Enlarged cardiac silhouette
Enlarged hilum
Hiatal hernia

Pneumothorax
Pleural abnormality

Consolidation
Groundglass opacity
Atelectasis
Lung nodule or mass
Pulmonary edema
High lung volume / emphysema
Interstitial lung disease

Acute fracture

Other

Drawing bounding boxes for Pulmonary edema is challenging, but attempt to include the key features of peribronchial cuffing and septal lines / interlobular septal thickening as best as possible. If diffuse and severe, the bounding boxes may encompass most of the lungs.

Support devices

Abnormal mediastinal contour
Enlarged cardiac silhouette
Enlarged hilum
Hiatal hernia

Pneumothorax
Pleural abnormality

Consolidation
Groundglass opacity
Atelectasis
Lung nodule or mass
Pulmonary edema
High lung volume / emphysema
Interstitial lung disease

Acute fracture

Other

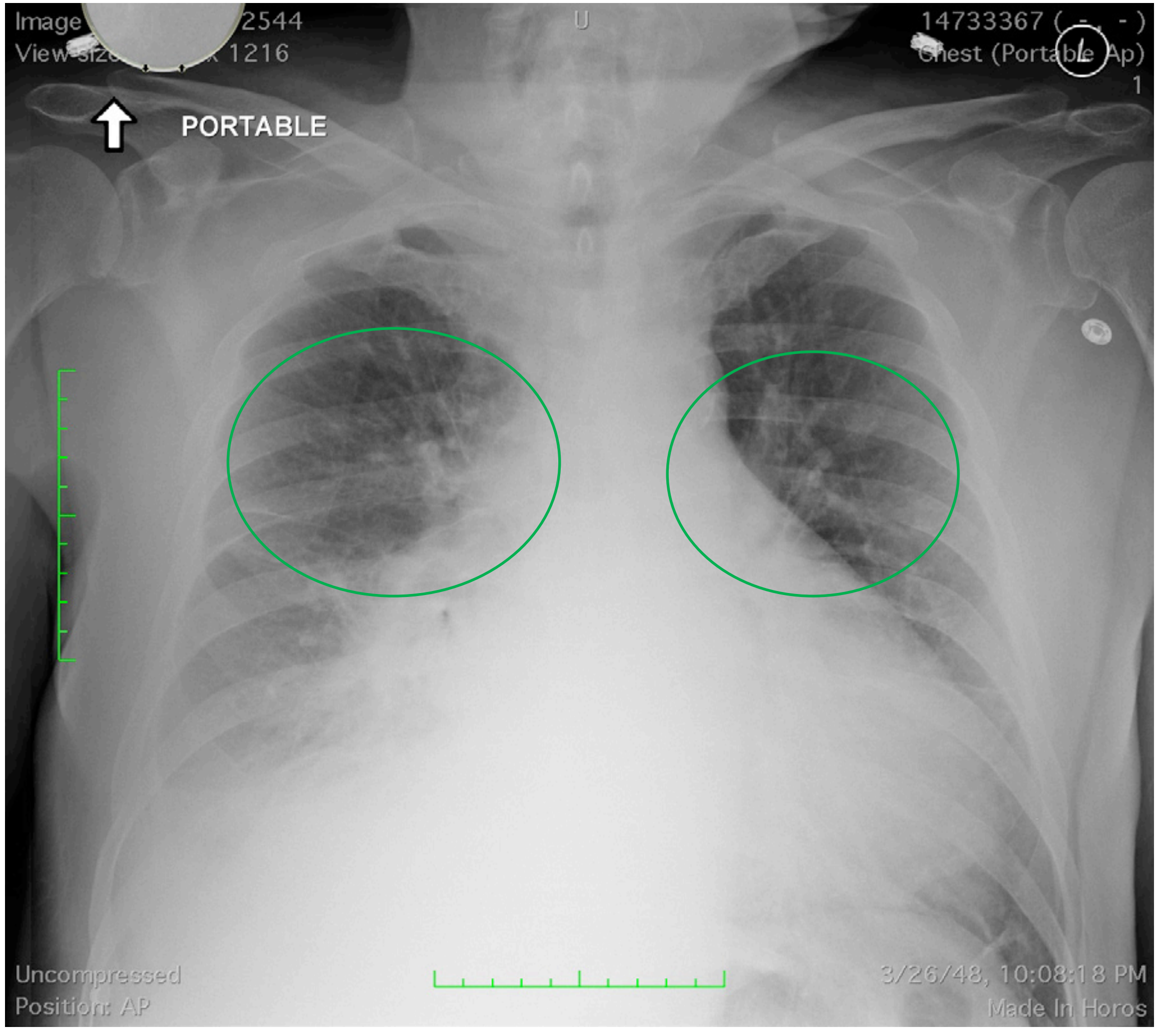

Support devices

Abnormal mediastinal contour
Enlarged cardiac silhouette
Enlarged hilum
Hiatal hernia

Pneumothorax
Pleural abnormality

Consolidation
Groundglass opacity
Atelectasis
Lung nodule or mass
Pulmonary edema
High lung volume / emphysema
Interstitial lung disease

Acute fracture

Other

Drawing bounding boxes for High lung volume / emphysema is challenging, but attempt to include the key features of flattened hemidiaphragms, typically upper lobe predominant lucencies and vascular pruning as best as possible. The bounding boxes may encompass most of the lungs.

Support devices

Abnormal mediastinal contour
Enlarged cardiac silhouette
Enlarged hilum
Hiatal hernia

Pneumothorax
Pleural abnormality

Consolidation
Groundglass opacity
Atelectasis
Lung nodule or mass
Pulmonary edema
High lung volume / emphysema
Interstitial lung disease

Acute fracture

Other

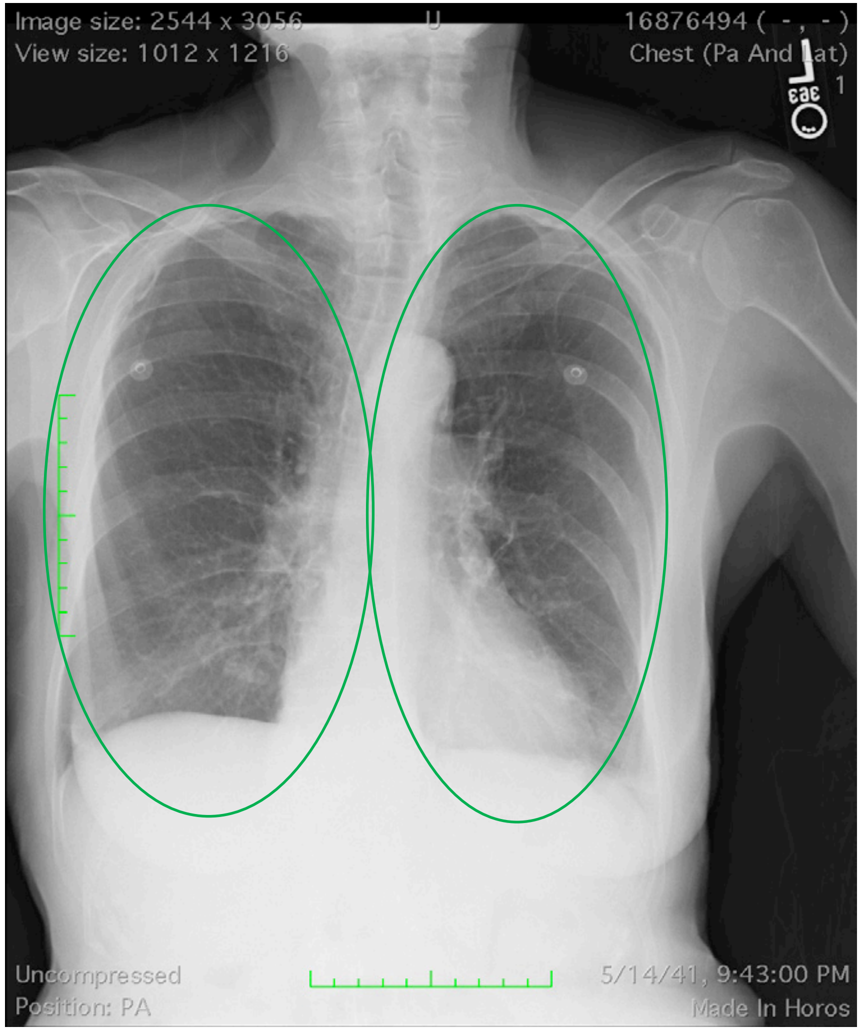

Support devices

Abnormal mediastinal contour
Enlarged cardiac silhouette
Enlarged hilum
Hiatal hernia

Pneumothorax
Pleural abnormality

Consolidation
Groundglass opacity
Atelectasis
Lung nodule or mass
Pulmonary edema
High lung volume / emphysema
Interstitial lung disease

Acute fracture

Other

Interstitial lung disease refers to diffuse lung disease, often peripheral and basilar predominant (e.g., UIP/IPF). Diffuse lung disease or the idiopathic interstitial pneumonias (IIPs) would typically be moderate to severe in extent to be identified on a chest radiograph. Use the bounding boxes to include typical features such as traction bronchiectasis and honeycombing. If perihilar abnormalities are present that are suggestive of other diffuse lung diseases (e.g., pneumoconiosis or sarcoidosis), we will also use the ‘interstitial lung disease’ label for these cases.

Support devices

Abnormal mediastinal contour
Enlarged cardiac silhouette
Enlarged hilum
Hiatal hernia

Pneumothorax
Pleural abnormality

Consolidation
Groundglass opacity
Atelectasis
Lung nodule or mass
Pulmonary edema
High lung volume / emphysema
Interstitial lung disease

Acute fracture

Other

Bounding boxes will be used to indicate Acute fracture. Chronic fractures of the ribs, clavicles, etc. do not need to be labeled.

Support devices

Abnormal mediastinal contour
Enlarged cardiac silhouette
Enlarged hilum
Hiatal hernia

Pneumothorax
Pleural abnormality

Consolidation
Groundglass opacity
Atelectasis
Lung nodule or mass
Pulmonary edema
High lung volume / emphysema
Interstitial lung disease

Acute fracture

Other

The Other label will be used for other clinically / radiologically significant features that in the radiologist’s opinion would typically be dictated in the report. Examples include pneumomediastinum, subcutaneous emphysema, and bronchiectasis.

Bounding boxes

If one region contains evidence related to one of several possible labels, select one bounding box containing all possible labels.

For example, if either Atelectasis **or** Consolidation is present in a specific part of the image, select them both in a single bounding box.

When choosing the certainty, select the one that reflects the odds of at least one of the chosen labels being correct. For example, if you are 95% certain that either Atelectasis or Consolidation is present, select “Consistent with (>90%).”

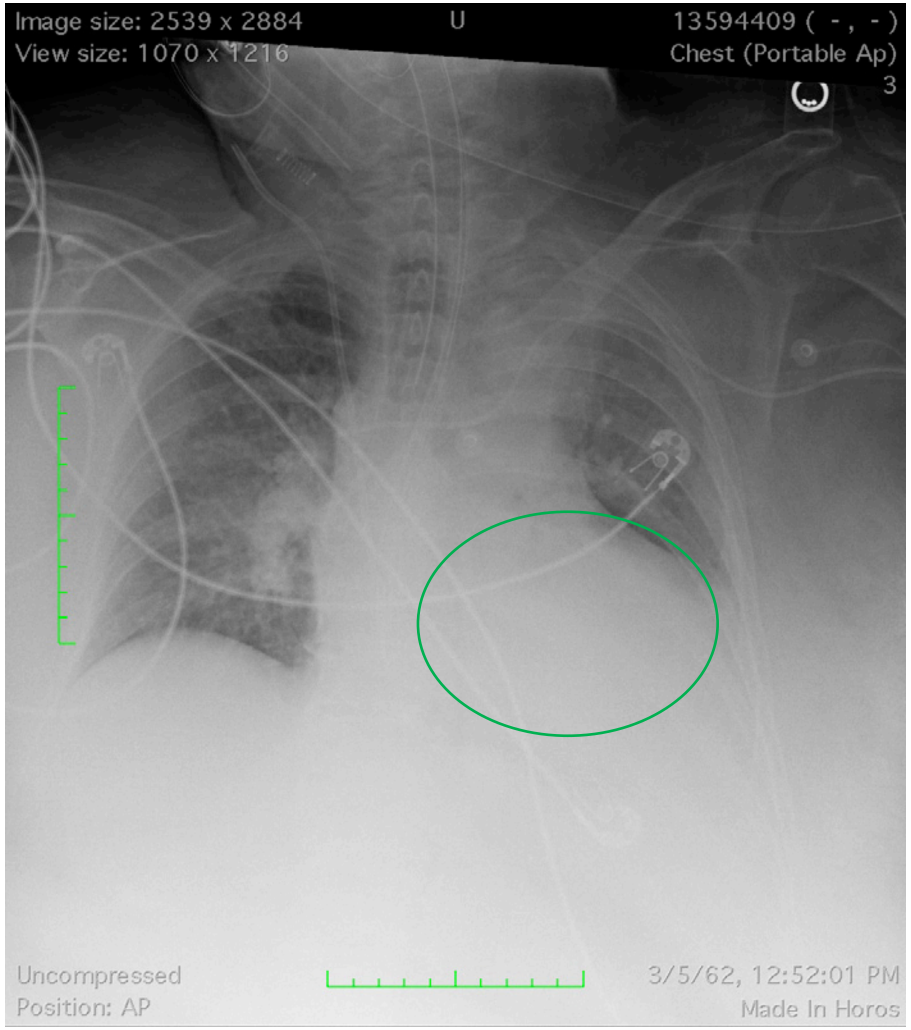

Bounding boxes

If one region contains evidence related to **all** of several labels (e.g., evidence for Enlarged cardiac silhouette and Hiatal hernia is present in that part of the image), select one box for each label.

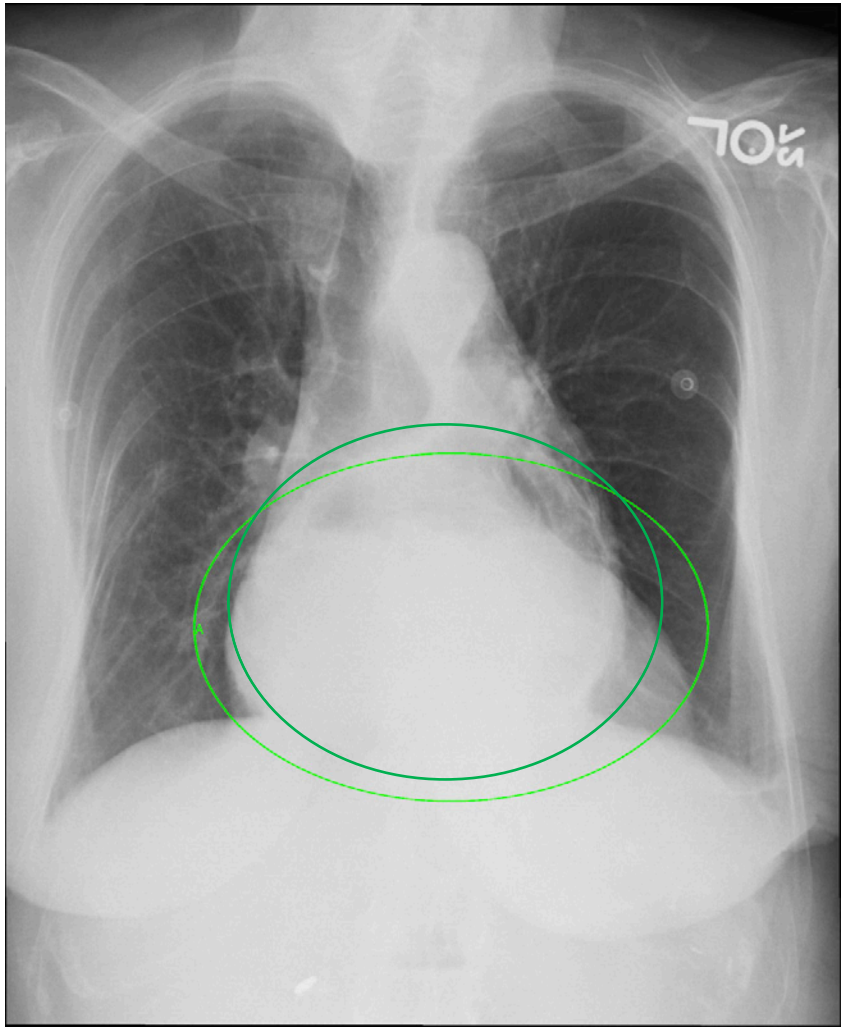

Certainty definitions, as defined by Panicek and Hricak (2016)[3]

Consistent with (>90%)

Suspicious for/Probably (~75%)

Possible/Possibly (~50%)

Less likely (~25%)

Unlikely (<10%)

Certainty definitions, as defined by Panicek and Hricak (2016)[3]

Consistent with (>90%)

Suspicious for/Probably (~75%)

Possible/Possibly (~50%)

Less likely (~25%)

Unlikely (<10%)

Consistent with (>90%):

"The proposed diagnosis is the best explanation for the imaging findings in view of the clinical information available, although a different diagnosis could be offered in different clinical circumstances."[3]

Certainty definitions, as defined by Panicek and Hricak (2016)[3]

Consistent with (>90%)

Suspicious for/Probably (~75%)

Possible/Possibly (~50%)

Less likely (~25%)

Unlikely (<10%)

Suspicious for/Probably (~75%):

“The proposed diagnosis is suspected on the basis of the imaging findings, but the findings are not pathognomonic for that diagnosis.” [3]

Certainty definitions, as defined by Panicek and Hricak (2016)[3]

Consistent with (>90%)

Suspicious for/Probably (~75%)

Possible/Possibly (~50%)

Less likely (~25%)

Unlikely (<10%)

Possible/Possibly (~50%):

“Some, but not all, of the imaging findings usually associated with the proposed diagnosis are present. Other findings not typically encountered in that diagnosis may be present.” [3]

Certainty definitions, as defined by Panicek and Hricak (2016)[3]

Consistent with (>90%)

Suspicious for/Probably (~75%)

Possible/Possibly (~50%)

Less likely (~25%)

Unlikely (<10%)

Less likely (~25%):

"The proposed diagnosis is believed to have a low likelihood of being correct but remains a plausible explanation for the imaging findings." [3]

Certainty definitions, as defined by Panicek and Hricak (2016)[3]

Consistent with (>90%)

Suspicious for/Probably (~75%)

Possible/Possibly (~50%)

Less likely (~25%)

Unlikely (<10%)

Unlikely (<10%):

"The proposed diagnosis is believed to have a low likelihood of being the actual explanation for the imaging findings." [3]

Support devices

Abnormal mediastinal contour
Enlarged cardiac silhouette
Enlarged hilum
Hiatal hernia

Pneumothorax
Pleural abnormality

Consolidation
Groundglass opacity
Atelectasis
Lung nodule or mass
Pulmonary edema
High lung volume / emphysema
Interstitial lung disease

Acute fracture

Other

Labeling screen:

Considering the list of labels made available to you, you should select even the ones you forgot to include in your dictation. It would be best to read every label from the list to check each of them individually.

Transcription editing screen:

Use this screen to correct only transcription errors and not to change the content of your dictation.

References

1. Johnson AEW et al. (2019) MIMIC-CXR, a de-identified publicly available database of chest radiographs with free-text reports. Scientific Data 6:317.
2. Hansell et al. (2008), Fleischner Society: Glossary of terms for thoracic imaging. Radiology 246(3):697-722.
3. Panicek and Hricak (2016), How sure are you, doctor? A standardized lexicon to describe the radiologist's level of certainty. AJR 207:2-3.
